# The effect of low insurance reimbursement on quality of care for non-small cell lung cancer in China: a comprehensive study covering diagnosis, treatment, and outcomes

**DOI:** 10.1186/s12885-018-4608-y

**Published:** 2018-06-25

**Authors:** Xi Li, Qi Zhou, Xinyu Wang, Shaofei Su, Meiqi Zhang, Hao Jiang, Jiaying Wang, Meina Liu

**Affiliations:** 1 Department of Biostatistics, School of Public Health, Harbin Medical University, Harbin, China; 2 School of Public Health, Harbin Medical University, No.157 Baojian Road, Harbin, 150081 China

**Keywords:** Insurance reimbursement rate, Non-small cell lung cancer, Quality indicators, Diagnosis, treatment, and outcomes

## Abstract

**Background:**

The insurance reimbursement rate of medical cost affects the quality and quantity of health services provided in China. The nature of this relationship, however, has not been reliably described in the field of non-small cell lung cancer (NSCLC). The objective of the current study was to examine the impact of low reimbursement rates of medical costs on diagnosis, treatment and outcomes among patients with NSCLC.

**Methods:**

We examined care of 2643 NSCLC patients and we divided the study cohort into a high reimbursement rate group and a low reimbursement rate group. The impact of reimbursement rates of medical costs on quality of care of NSCLC patients were examined using logistic regression and generalized linear models.

**Results:**

Compared with patients insured with high reimbursement rate, patients insured through lower reimbursement rate programs were less likely to benefit from early detection and treatment services. Delayed detection was more common in low reimbursement group and they were less likely to be recommended for adjuvant chemotherapy, or to receive adjuvant chemotherapy and postoperative radiation therapy and they had lower odds to receipt chemotherapy response assessment. However, low reimbursement rate group had lower rate of in-hospital mortality and metastases.

**Conclusions:**

Low reimbursement rate mainly negatively influenced the diagnosis and treatment of NSCLC. Reducing the gap in reimbursement rate between the three health insurance schemes should be a focus of equalizing access to care and improving the level of medical compliance and finally improving quality of care of NSCLC.

**Electronic supplementary material:**

The online version of this article (10.1186/s12885-018-4608-y) contains supplementary material, which is available to authorized users.

## Background

Insurance is a significant determinant of access to health care and, consequently, of high quality of care. The level of insurance reimbursement of medical costs plays a vital role in determining the quality and quantity of health services provided [1–6]. Health insurance, a mutual help and risk-pooling health protection system, generally does not cover health care costs in full. The primary payer status varies, with different insurance types having markedly different deductibles, copays, and reimbursement caps. Insurance and the alleviation of cost-related barriers to health care have achieved tremendous progress in the prevention, early detection, and high-quality treatment of cancer. However, this has not been experienced equally by all segments of the insured population, and individuals insured with lower reimbursement rates may be disadvantaged.

Many developing countries have begun to establish and implement universal health coverage. China essentially achieved this goal by the end of 2011. China’s health insurance system is a combination of compulsory and voluntary insurance types. It primarily consists of three basic social health insurance programs, which are uniformly government-supported and cover more than 95.7% of the Chinese population [7]. The programs have their own defined target populations, premiums, benefit programs, and implementation guidelines [8]. New Rural Cooperative Medical Scheme (NCMS) is designed for the rural population. Its enrollment covers 62% of the Chinese population. Urban Resident Basic Medical Insurance (URBMI) targets the unemployed, children, the disabled, and elderly people in urban areas, and Urban Employed Basic Medical Insurance (UEBMI) is for urban employees. UEBMI covers 19% of the population, and URBMI covers 16% [9]. Insurance mainly pays for in-hospital care. The reimbursement rate for NCMS is 50–65%—much lower than UEBMI’s rate of 85–95% but similar to URBMI’s rate of 50% [6].

Much attention has been paid to the effect of insurance status on quality of care [10–15], but few studies have focused on the effect of a critical attribute of insurance—reimbursement rate [5, 6]. Past work has analyzed the relationship between insurance status and quality of care for non-small cell lung cancer (NSCLC) [16–18], mostly focusing on limited aspects such as clinical treatment or subsequent progress. For example, Potosky and colleagues examined the impact of insurance status on the initial treatment of NSCLC [19], and Bradley et al. analyzed cancer diagnosis and survival disparities by insurance types [20]. Few studies have investigated the whole process from NSCLC diagnosis, to treatment, to prognosis using process-of-care and outcome indicators, and no studies have evaluated the effect of reimbursement rate on quality of care for NSCLC. Thus, this study aimed to explore the influences of a lower-rate reimbursement program for patients with NSCLC throughout the process, including preoperative diagnosis, treatment, and postoperative outcomes.

## Methods

### Study cohort

**Fig. 1:**
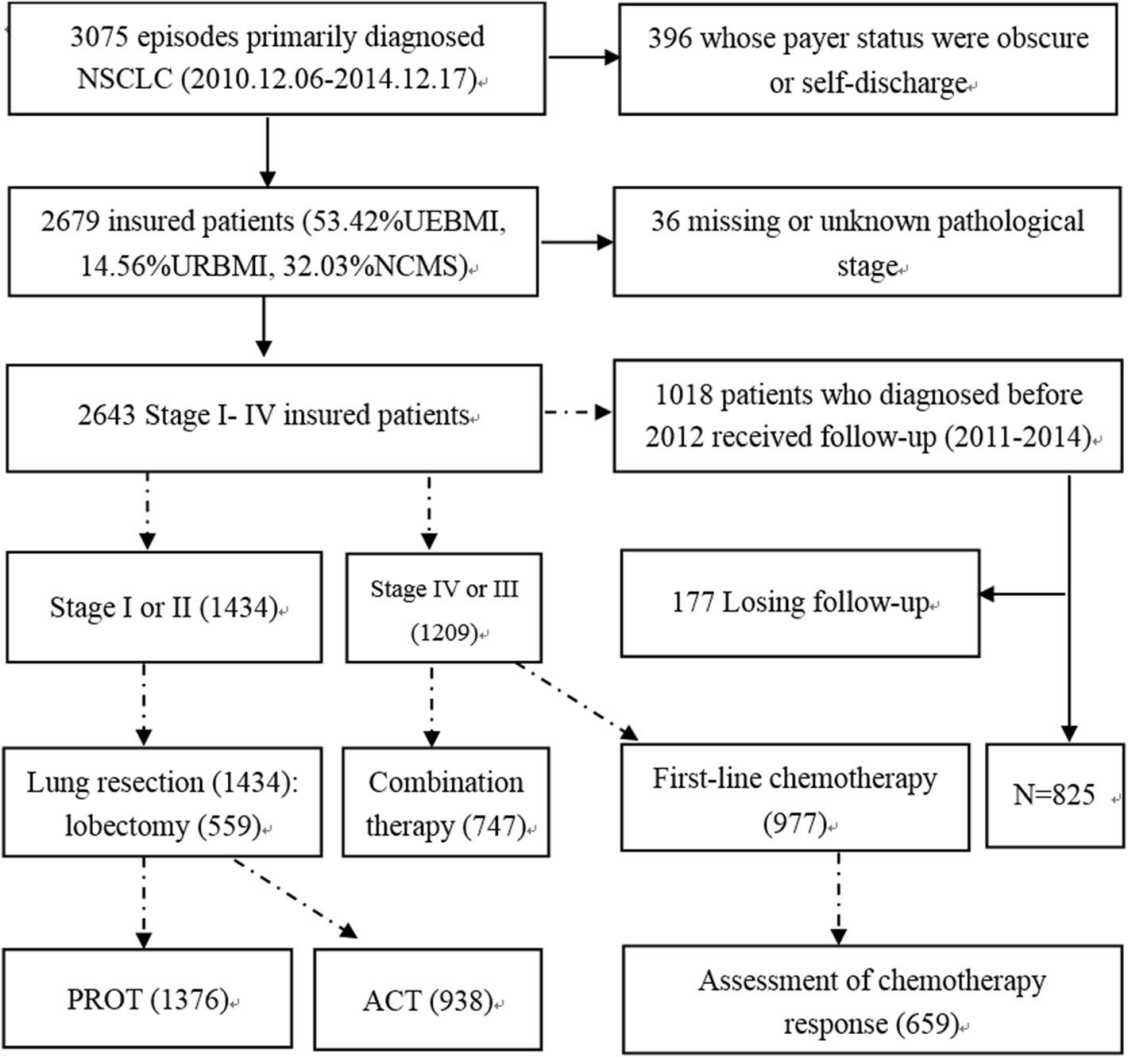
“Solid line” means study flow diagram of the patient population. “Dotted line” means flowchart for treatments and follow-up group. The number in parentheses represents the sum of patients eligible for the evidence-based care, due to the limited space, we only showed the stage related care and its eligible population size. Abbreviations: NSCLC: non-small cell lung cancer, NCMS: New Rural Cooperative Medical Scheme, URBMI: Urban Resident Basic Medical Insurance, UEBMI: Urban Employed Basic Medical Insurance, ACT: Adjuvant chemotherapy, PORT: postoperative radiation therapy

This study was part of research fields of our research group to evaluate the quality of care for breast, colorectal, and lung cancers. After receiving the approval of the medical institutional records directors at each site, we obtained the medical records of all patients meeting the inclusion criteria. Patients who received initial examinations and treatment at other facilities before receiving inpatient treatment at the selected hospitals remained eligible for the study. From the available pool of eligible patients primarily diagnosed with NSCLC, we excluded 57 patients who were unwilling or unable to consent and identified a study cohort of 3075 individuals aged 18–70 with a primary diagnosis of NSCLC made from 6 December 2010 to 17 December 2014 who underwent inpatient treatment for stage I–IV cancer in the selected hospitals. Follow-up was conducted with those patients diagnosed before 2012 through facility visits and telephone calls. This follow-up began two to 4 weeks after the patients left the hospital and was repeated every 3 months for 2 years. Patients outside the age range, those who received only outpatient care, and those who also had other malignant tumors or mixed small-cell lung cancer were excluded from the study. Because this study aimed to analyze the influence of low reimbursement rates on quality of care for NSCLC, patients with obscure primary payer status and those who self-discharged were not included in the study. The final analytical sample comprised 2643 insured patients who received inpatient treatment for stage I–IV NSCLC. Fig. 1 presents the number of study flow diagram of the patient population.

### Data collection

A questionnaire for NSCLC cases was drafted by a team of oncology professionals, clinical physicians, and epidemiologists. The questionnaire (see Additional file 2) gathered routinely collected medical information on several domains: patient demographics, tumor characteristics, diagnosis, NSCLC treatment and prognosis, and information necessary for identifying eligible patients for evidence-based care. Data on primary payer status were collected as part of the patient demographics. Before the data collection, data abstractors received 3 weeks of training organized by oncology professors and the principal investigators. Information extraction was performed systematically, following the operations manual. To guarantee the validity and reliability of the questionnaire, we conducted a pilot test. During the data collection process, regular correspondence was maintained with those compiling the data to identify any ambiguities or deficiencies in the information collection to facilitate timely modification and accelerate the process of data extraction. Following the data collection, 5% of the records were randomly selected for a secondary data collection using methods identical to the first data collection, and the test-retest reliability was high (up to 95%).

### Patient demographics

Baseline demographic information abstracted from the medical history records included age group (< 50, 50–60, ≥ 60), gender, primary payer status (NCMS, URBMI, or UEBMI), household income, smoking, comorbidities, and postoperative clinical report information. According to the disparities of reimbursement rate among insurance type, we divided the study cohort into two payer groups, including a high reimbursement rate group (UEBMI) and a low reimbursement rate group (URBMI and NCMS). Per capita annual income was derived from the bulletin of social development published by the statistical bureau. The national average annual income from 2011 to 2014 was used to divide the patients into two groups (low-income and high-income). We also calculated an Charlson comorbidity index (CCI: 0, 1 to 3, ≥ 4), a weighted index of 16 conditions found to significantly influence prognosis among cancer patients, with scores assessed based on relative mortality risk. Patients were considered to have a comorbid condition if a listed disorder was mentioned in their medical or treatment-related records. Institutional Research Board of Harbin Medical University approved the study and written informed consent was obtained from all individual participants included in the study.

### Tumor characteristics

Lung cancer-specific information assessed for each patient included primary lesion site, tumor size, histological grade, histological classification (adenocarcinoma, squamous cell carcinoma, other), tumor stage (I–IV), distant metastases, and bronchial stump. Variables with more than 5% missing data ware regarded as “unknown.” Otherwise, missing data were taken as real missing data. However, there were some deficiencies in the medical records, mainly in tumor stage, which included incorrect or incomplete information. Given the significance of stage information for identifying eligible patients for a certain clinical treatment, we filled in the missing information and corrected errors by consulting oncologists and pathologists and through the joint effort of our team based on the condition of the primary tumor, lymphatic metastasis, and distant metastasis of the patients and using the international Tumor-Node-Metastasis (TNM) classification system [21].

### Dependent variables

The research team selected 11 priority process-of care measures based on the evidence-based guidelines of recommended care, established associations between care and outcomes, relatively independent of each indicator, and data integrity. This selection included the diagnostic and treatment process and was developed by our research group through consulting many references and conducting a three-round modified Delphi panel process. The selected measures were skeletal scintigraphy and brain Magnetic Resonance Imaging (MRI) or Computed Tomography (CT), pulmonary function test (PFT), epidermal growth factor receptor gene mutation test, adjuvant chemotherapy (ACT), recommendation for ACT, postoperative radiation therapy (PORT), radiographic assessment of chemotherapy response, first-line chemotherapy, lobectomy, surgical resection, and combination therapy. Each process-of-care indicator was defined by its inclusion or exclusion criteria according to the standard eligibility definition (see Additional file 1). Considering suspected universal adherence, postoperative pathological report and electrocardiogram were removed. In addition, because of data incompleteness (close to 50% missing) or insufficient eligible patients, performance status assessment and neoadjuvant chemotherapy were excluded from our research. Figure 1 presents the flowchart for the main treatments.

Five quality-of-care measures were also selected as outcomes of interest in this study: postoperative complications, metastases, in-hospital mortality, 2-year fatality rate, and length of hospital stay.

### Primary payer status

Primary payer status was routinely recorded in patient discharge records. In cases where payer status information was missing here, the medical records home page could alternatively be reviewed to find the information. In the few cases where payer status was missing from both locations, it was treated as “unknown.” Self-discharge patients were excluded because of ambiguity regarding payer status; in these patients’ records, uninsured patients, commercially insured patients, and even those with multiple insurance coverage were merged. In addition, other patients with indeterminate payer status information were also excluded from the study.

### Statistical analysis

Descriptive statistics were used to compare baseline characteristics and the utilization of the 16 process-of-care and outcome-of-care indicators by primary payer status. We calculated the number of eligible cases for each individual measure in each payer group. Utilization of each indicator was calculated using the sum of patients receiving care as the numerator and the sum of patients eligible for that type of care as the denominator. Composite performance scores were calculated using opportunity-based scores, defined as the sum of eligible patients who actually received care divided by total care opportunities [22]. Simple bivariate comparisons were conducted with Chi-squared or Kruskal–Wallis H tests, depending on the variable type.

Separate regression models were used for each measure. Individual and tumor characteristics, as well as hospital category, were selected as covariates that potentially influence primary care experiences and the incidence of particular outcomes. Multivariate logistic regression models were used to examine the independent effects of insurance type on treatment and outcome by controlling for these confounding effects. Because the variables were not normally distributed, the association between length of stay and insurance type was analyzed using generalized linear models with a gamma distribution and log link function. The odd ratios (ORs) and their 95% confidence intervals were estimated. Concordance indexes were calculated to determine model diagnostics, providing an estimate of the predictive accuracy of the models. A value of 0.5 demonstrates that outcomes are completely random, whereas a value of 1 demonstrates the perfect predictive accuracy of the model. All data were analyzed anonymously. All analyses were performed using SAS version 9.3.1 (SAS Institute, Cary, NC) and used two-tailed tests of statistical significance, with the significance level set at *P* < 0.05.

## Result

### Baseline demographic information and tumor characteristics

Of the sample of 2643 patients, 1419 (53.69%) were covered by insurance with high reimbursement rate and 1224 (46.31%) were covered by insurance with low reimbursement rate. Over half of the patients were diagnosed with stage I or II NSCLC, and 56% received treatment at specialized tumor hospitals. Non-squamous cell histology was observed in 63.83% (1687 in 2643) of the patients, and the majority of these cases were adenocarcinoma (1344 in 1687). With respect to socioeconomic status, less than one-fifth of the patients earned over the national average annual income.

There were variations in the baseline demographic data and tumor characteristics of NSCLC patients who were insured with low reimbursement rate versus insured with high reimbursement rate. Of the 12 variables examined, statistically significant variations were observed in 10. In comparison with high reimbursement group, patients insured through low reimbursement rate programs had a similar primary lesion site, similar proportion of smokers and incidence rate of positive bronchial stump. Low reimbursement rate group were less likely to have family history of NSCLC (4.41% vs. 6.69%), to complicate other diseases (CCI = 0, 23.12% vs. 14.59%), but they were younger to suffer from NSCLC (age < 50, 24.67% vs. 15.86%), more likely to be diagnosed in a later stage (stage III- IV, 47.63% vs. 43.11%), to be diagnosed with low differentiated carcinoma (32.43% vs. 26.15%), and to have lower socioeconomic status (high income, 4.00% vs. 29.32%). Details of patients’ demographic data and tumor characteristics by primary payer status are listed in Table 1.

**Table 1:** Baseline demographic and tumor characteristics by primary payer status^a^

Characteristics	Overall *n* (%)	High reimbursement rate, *n* (%)	Low reimbursement rate, *n* (%)	*P*
CCI				
0	490(18.54)	207(14.59)	283(23.12)	<.0001
1~ 3	2085(78.89)	1174(82.73)	911(74.43)	
4~	68(2.57)	38(2.68)	30(2.45)	
Gender				
male	1677(63.45)	939(66.17)	738(60.29)	0.0018
female	966(36.55)	480(33.83)	486(39.71)	
Age				
< 40	82(3.10)	33(2.33)	49(4.00)	<.0001
40~	445(16.84)	192(13.53)	253(20.67)	
50~	1083(40.98)	600(42.28)	483(39.46)	
60~	1033(39.08)	594(41.86)	439(35.87)	
Smoking				
no	1174(44.42)	631(44.47)	543(44.36)	0.9567
yes	1469(55.58)	788(55.53)	681(55.64)	
Family history of NSCLC				
none	2494(94.36)	1324(93.31)	1170(95.59)	0.0112
have	149(5.64)	95(6.69)	54(4.41)	
primary lesion site				
left	1051(39.77)	560(39.46)	491(40.11)	0.9437
right	1416(53.58)	764(53.84)	652(53.27)	
other	176(6.66)	95(6.69)	81(6.62)	
Historical stage				
High differential	302(11.27)	189(13.32)	112(9.15)	<.0001
Moderately differential	710(26.50)	412(29.03)	294(24.02)	
Low differential	779(29.08)	371(26.15)	397(32.43)	
unknown	868(32.84)	447(31.50)	421(34.40)	
Histological classification				
Squamous carcinoma	956(36.17)	483(34.04)	437(38.64)	0.0063
adenocarcinoma	1334(50.47)	759(53.35)	577(47.14)	
other	353(13.36)	179(12.61)	174(14.22)	
Procedure class				
lobectomy	1576(59.63)	876(61.73)	210(55.56)	0.0049
wedge resection	67(2.53)	45(3.17)	6(1.59)	
pneumonectomy	229(8.66)	104(7.33)	34(8.99)	
exploratory thoracotomy	771(29.17)	394(27.77)	128(33.86)	
Bronchial stump				
negative	1696(64.17)	923(65.05)	773(63.15)	0.5386
positive	43(1.63)	24(1.69)	19(1.55)	
unknown	904(34.20)	472(33.26)	432(35.29)	
Clinical stages				
IA	559(21.15)	333(23.47)	226(18.46)	0.0065
IB	426(16.12)	213(15.01)	213(17.40)	
IIA	325(12.30)	183(12.90)	142(11.60)	
IIB	124(4.69)	64(4.51)	60(4.90)	
IIIA	607(22.97)	301(21.21)	309(25.00)	
IIIB	147(5.56)	71(5.00)	76(6.21)	
IV	455(17.22)	254(17.90)	201(16.42)	
Hospital type				
Specialized	1480(56.00)	741(52.22)	739(60.38)	<.0001
General	1163(44.00)	678(47.78)	485(39.62)	
Average per capital income				
High income	465(17.59)	416(29.32)	49(4.00)	<.0001
Low income	2178(82.41)	1003(70.68)	1175(96.00)	

^a^Data are expressed as numbers and percentages of patients. Percentages may not sum up to 100% due to round-off.Abbreviations: *CCI* the Charlson comorbidity index, *NSCLC* non-small cell lung cancer

### Disparities in utilization of NSCLC treatment process and outcomes by primary payer status

Composite performance scores for the NSCLC process of treatment and outcome didn’t vary significantly by primary payer status (Table 2). The unadjusted adherence or incidence of each indicator by primary payer status is shown in Table 3. Compared with patients insured with high reimbursement rate, underutilization of process-of-care indicators was found among patients insured with low reimbursement rate, who had comparatively lower probability for being recommended for ACT (37.96% vs. 48.26%, *P* = 0.0187) or receiving ACT (44.69% vs. 52.24%, *P* = 0.0484), PORT (0.49% vs. 2.88%, *P* = 0.0010) or radiographic assessment of chemotherapy response (47.02% vs. 59.41%, *P* = 0.0014). A high level of PFTs were given to patients insured with low reimbursement rate, with a receipt rate approaching 87.85%. Regarding disparities in outcomes, in-hospital mortality (1.47% vs. 3.66%, *P* = 0.0005) and metastases rates (8.09% vs. 10.75%, *P* = 0.0488) were lower in patients insured with low reimbursement rate. Of all surgical patients, 5.53% developed complications and 9.65% of patients had metastases; there were no statistically significant difference in 2-year mortality by payer status (*P* = 0.2862). The mean total length of hospital stay was 21.11 days (standard deviation [SD] = 16.76) and was similar across payer statuses (*P* = 0.0672) but the length of preoperative hospital stay varied (*P* < 0.0001).

**Table 2:** Adherence to composite indicator by payer status^a^

composite indicator	High reimbursement rate			Low reimbursement rate			*P*
	M	N (%)		M	N (%)		
Process	5463	3226 (59.05)		4611	2714(58.86)		0.8448
Outcome	3881	293(7.55)		3419	243(9.36)		0.4697

^a^“M” means the sum of total patients who were eligible and have none of the contraindications for each indicator, “N” means eligible patients who were actually received the treatment, the percentile in parentheses represents composite score of process-of-care indicators and outcome indicator according to payer status

**Table 3:** Unadjusted adherence to quality-of-care indicators by payer status (%)^a^

Indicators (No. eligible)	Overall	High reimbursement rate	Low reimbursement rate	*P*
ECT and brain MRI or CT (752)	57.58	60.92	54.33	0.0677
PFTs (1909)	81.72	76.65	87.85	<.0001
EGFR mutation test (453)	3.31	4.76	1.49	0.0533
ACT (938)	48.84	52.24	44.69	0.0484
Recommended for ACT (533)	44.09	48.26	37.96	0.0187
PORT (1376)	1.82	2.88	0.49	0.0010
ACT response assessment (659)	53.41	59.41	47.02	0.0014
First-line chemotherapy (977)	69.54	68.60	70.56	0.5087
Lobectomy (559)	84.97	83.18	87.61	0.1505
Surgical resection (1434)	96.16	96.85	95.32	0.1342
Combination therapy (747)	61.58	60.87	62.27	0.6942
Complications (1916)	5.53	5.42	5.66	0.8181
Metastases (1916)	9.65	10.75	8.09	0.0488
In-hospital mortality (2643)	2.65	3.66	1.47	0.0005
2-year mortality rate (825)	21.45	19.72	22.80	0.2862
total length of hospital stay (2643)	21.11 ± 16.76	21.30 ± 16.56	20.89 ± 17.00	0.0672
preoperative length of hospital stay (1916)	7.56 ± 6.55	7.84 ± 6.27	7.22 ± 6.86	<.0001

^a^Discrete variables were expressed as counts (%) and continuous variables were expressed as a mean ± range. *Abbreviations*: *ECT* and brain *MRI* or *CT* skeletal scintigraphy and brain magnetic resonance imaging or computed tomography, *PFTS* pulmonary function tests, *EGFR* epidermal growth factor receptor, *ACT* adjuvant chemotherapy, *PORT* postoperative radiation therapy

**Fig. 2:**
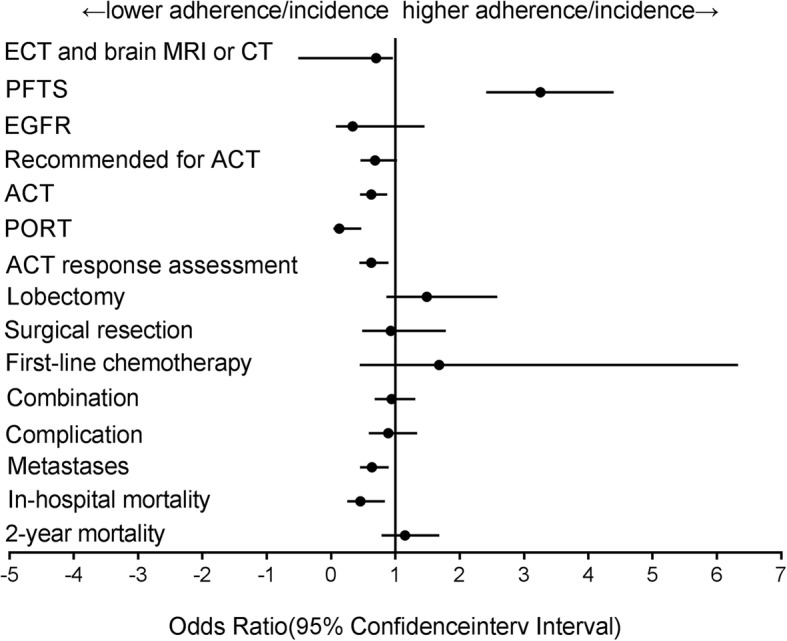
Adjusted adherence to quality indicators and incidence of adverse outcome in lower reimbursement rate group compare with higher reimbursement rate group (OR, 95%CI). All indicators uniformly adjusted for ACCI, gender, smoking, family history of NSCLC, average per capital income, historical stage, histological classification, pathological stage, hospital type. Outcome indicators additionally adjusted procedure class. Abbreviations: ECT and brain MRI or CT: skeletal scintigraphy and brain Magnetic Resonance Imaging or Computed Tomography, PFTS: pulmonary function tests, EGFR: epidermal growth factor receptor, ACT: Adjuvant chemotherapy, PORT: postoperative radiation therapy

Figure 2 present the results for adjusted adherence to quality indicators and incidence of adverse outcomes by payer status. The majority of types of recommended care were underused among patients insured through the lower reimbursement rate program. After adjusting for patients’ demographic and tumor characteristics, low reimbursement rate group were less likely to have skeletal scintigraphy and brain MRI or CT (OR = 0.701, 95%CI 0.510–0.962), or to receive ACT (OR = 0.627, 95%CI 0.450–0.873), PORT (OR = 0.129, 95%CI 0.036–0.469) and radiographic assessment of chemotherapy response (OR = 0.627, 95%CI 0.441–0.893) than high reimbursement rate group. As for the outcome, low reimbursement rate group were less likely to die in the hospital (OR = 0.458, 95%CI 0.250–0.837) or have postoperative metastases (OR = 0.635, 95%CI 0.450–0.897) than high reimbursement group, but there was no significant difference of 2-year mortality risk between groups. The comparison of the total and preoperative length of hospital stay by primary payer status is displayed in Table 4. No marked differences were found in the preoperative length of hospital stay by payer status, but the length of total stay did differ significantly after adjusting for confounding variables.

**Table 4:** Preoperative and total length of hospital stay for NSCLC patients hospitalized for surgical care by payer status^a^

Variables	Coefficient	SE	*waldχ* ^2^	*P*
total length of hospital stay				
High vs Low	−0.1173	0.0335	12.26	0.0005
preoperative length of hospital stay				
High vs Low	−0.0351	0.0584	0.36	0.5475

^a^Adjusted for CCI, age, gender, family history of NSCLC, average per capital income, historical stage, histological classification, pathological stage, hospital type, procedure class. *Abbreviations*: *CCI* the Charlson comorbidity index, *NSCLC* non-small cell lung cancer

## Discussion

The impact of primary payer status on quality of care for NSCLC was comprehensively assessed from diagnosis, to treatment, to outcome, using 11 process-of-care indicators and five outcome indicators. Using public health data, we established an association between primary payer status and quality of care that is of importance for both clinical and public health practice. The mean concordance indexes of the models was 0.76, indicating high discriminatory accuracy and the ability to make an accurate prediction. Although the results presented here were based on the insured population aged 18–70 with a primary diagnosis of NSCLC, the relevant population varied by model depending on the eligible population and the missing data or unobtainable values for each indicator. To obtain practical and targeted results, the pool of covariates for diagnosis, treatment, and outcome indicators were not identical across models. The covariates were selected based on clinical evidence-based correlations with each treatment.

After adjusting for patients’ demographic and tumor characteristics, clear disparities in NSCLC diagnosis and treatment were found by payer status. Patients insured through lower reimbursement rate programs were less likely to benefit from early detection and treatment services. These findings are in line with prior studies identifying negative effects of low reimbursement rates on diseases detection and treatment [5, 23, 24].

Non-adherence was associated with higher health care expenses [25]. As it is reported that medical expenses could account for non-compliance in 10% of patients [26]. The prepayment structure of health insurance schemes have intended to shift funds from the rich to the poor. But according to our results, patients insured with low reimbursement rate earned less actually paid more. Generally, an underutilization of clinically recommended care was found for patients insured with a low reimbursement rate, who were partly made up of rural-to-urban migrants or those referred from township or county-level hospitals. Lower reimbursement rates of medical costs signified higher out-of-pocket payments for patients, especially for the catastrophic expenditures required in cancer care [27]. This could undermine patients’ willingness to seek care. Reimbursement rates for patients covered by different insurance types varied by hospital type. NCMS funding generally requires patients to visit designated hospitals in their county. Although these patients qualify for the reimbursement of medical charges outside of their home counties, the rates are reduced dramatically [6, 28].This may directly cause a low adherence to treatment regimens and finally leads to interrupted or suspended treatment among this payer status group [29]. However, those covered by insurance with high reimbursement rate had almost equivalent reimbursement rates in all medical institutions, thus they could seek medical care at higher level medical institutions, which helps to ensure a relatively high quality of medical care.

Low incomes and inadequate reimbursement rates led to curtailed access. Many factors other than reimbursement rate are also likely to limit access to care. ACT was generally received by patients on day 30 after curative resection and then repeated at three-week intervals. Likewise, there are intervals in PORT. Under these circumstances, a long distance to the hospital, increased travel burdens, patient or family preferences, a lack of understanding of the importance of appropriate adjuvant therapy, and the unmeasured confounding of performance status may be barriers to adherence to treatment for patients insured with low reimbursement rate [30]. Because radiographic assessment of chemotherapy response is expensive and requires a high-level facility not found in township hospitals and limited reimbursement may undermine care-seeking behavior of patients insured with low reimbursement rate. There is an exception to the trend of underutilization among patients insured with low reimbursement rate: They have the highest adherence of PFTs. Future work should focus on specific aspects of recommendations for care, access to care, and delivery of care, incorporating integrated data. This may contribute to understanding the underlying mechanisms generating treatment disparities among NSCLC patients by primary payer status.

In contrast to previous studies [31, 32], we found that patients insured with low reimbursement rate have a lower rate of in-hospital mortality and metastases, and stayed shorter in the hospital; no significant negative influence of low reimbursement rate was found on 2-year mortality in this payer group. Except for the influences of low reimbursement rate of medical cost, a confounding influence may be found in the convention that “fallen leaves return to their roots—to revert to one’s origin”, because rural patients may refuse further therapy on their deathbed, choosing to die at home rather than in the hospital. Besides, facilities generally would not collect follow-up data on these patients, and this may have contributed to a low in-hospital mortality rate for patients insured with low reimbursement rate. Our mortality estimate for this group was somewhat lower than that found in prior research [19], because we used a treated and insured population consisting mostly of early stage and surgery (59.43% for lobectomy) patients [33–35]. The fact that insurance mainly reimburses for inpatient care that may contribute to shorter hospital stays among low reimbursement groups. No marked differences were found in length of preoperative hospital stay, implying similar preoperative waiting times across insurance types.

We provide an integrated appraisal of the effect of low reimbursement rates on the continuum of care for patients with NSCLC, including diagnosis, treatment, and outcome. The results were not perfectly in accordance with our expectations. Further study is required to explore the association between care and outcome. The identified disparities by primary payer status serve as an important proxy for the apparent cost-related barriers to health care among patients insured with low reimbursement rates and other health system-related issues. Non-adherence was associated with higher out-of-pocket expenses. Increased reimbursement rate for medical might be effective in securing good medical compliance. Our findings could provide support for health reforms on equalizing reimbursement rate, aiming at equalizing access to care and improving the level of medical compliance and finally improving quality of care of NSCLC.

Because of several limitations, caution must be exercised in interpreting the results of this study. First, we conducted observational research; therefore, we cannot prove causation between quality-of-care measures and insurance. Second, the hospitals participating in our study were exclusively tertiary teaching facilities located in urban areas, and this limits the generalizability. Future studies should also consider non-teaching, privately owned, community, and other classes of hospitals in a larger regional scope. Third, we did not analyze all established quality-of-care or confounding variables (e.g., distance from residence to hospital), and education levels were not adjusted in the multivariable analysis because of a large number of missing values. This may further limit the interpretation and generalizability of the results. Fourth, the follow-up time was too short to capture more significant differences in mortality. Different results may be obtained through continual tracking.

## Conclusion

We conducted univariate and multivariate analyses for a set of 16 quality-of-care indicators for NSCLC. The study found that low reimbursement rates had primarily negative influences on the diagnosis and treatment of NSCLC in patients. Patients insured through lower reimbursement rate programs were less likely to benefit from early detection and treatment services.

## Additional files

Additional file 1: **Table S1.** Eligible definition of selected indicators. (DOCX 15 kb)

Additional file 2: **Table S2.** Medical record questionnaire for non-small cell lung cancer patients. (DOCX 22 kb)

## Abbreviations

ACCI: Age-adjusted Charlson comorbidity index
ACT: Adjuvant chemotherapy
CT: Computed tomography
MRI: Magnetic resonance imaging
NCMS: New Rural Cooperative Medical Scheme
NSCLC: Non-small cell lung cancer
PFT: Pulmonary function test
PORT: Postoperative radiation therapy
UEBMI: Urban employed basic medical insurance
URBMI: Urban resident basic medical insurance

## References

[1] Hsia J, Kemper E, Kiefe C, Zapka J, Sofaer S, Pettinger M, Bowen D, Limacher M, Lillington L, Mason E (2000). The importance of health insurance as a determinant of cancer screening: evidence from the Women's Health Initiative.

[2] Institute of Medicine (US) Committee on the Consequences of Uninsurance. Care without Coverage: Too Little, Too Late [J]. J Natl Med Assoc. 2002;97(11):1578.

[3] Ward E, Halpern M, Schrag N, Cokkinides V, Desantis C, Bandi P, Siegel R, Stewart A, Jemal A (2008). Association of insurance with cancer care utilization and outcomes. CA Cancer J Clin.

[4] Skaggs DL, Lehmann CL, Rice C, Killelea BK, Bauer RM, Kay RM, Vitale MG (2006). Access to orthopaedic care for children with medicaid versus private insurance: results of a national survey. J Pediatr Orthop.

[5] Hagihara A, Murakami M, Chishaki A, Nabeshima F, Nobutomo K (2001). Rate of health insurance reimbursement and adherence to anti-hypertensive treatment among Japanese patients. Health Policy.

[6] Pan Y, Chen S, Chen M, Zhang P, Qian L, Li X, Lucas H (2016). Disparity in reimbursement for tuberculosis care among different health insurance schemes: evidence from three counties in Central China. Infect Dis Poverty.

[7] Meng Q, Xu L, Zhang Y, Qian J, Cai M, Xin Y, Gao J, Xu K, Boerma JT, Barber SL (2012). Trends in access to health services and financial protection in China between 2003 and 2011: a cross-sectional study. Lancet.

[8] Yip WC, Hsiao WC, Chen W, Hu S, Ma J, Maynard A (2012). Early appraisal of China's huge and complex health-care reforms. Lancet.

[9] Yu H (2015). Universal health insurance coverage for 1.3 billion people: what accounts for China's success?. Health Policy.

[10] Bradley CJ, Given CW, Roberts C (2003). Late stage cancers in a Medicaid-insured population. Med Care.

[11] Roetzheim RG, Pal N, Tennant C, Voti L, Ayanian JZ, Schwabe A, Krischer JP (1999). Effects of health insurance and race on early detection of Cancer. J Natl Cancer Inst.

[12] Harlan LC, Greene AL, Clegg LX, Mooney M, Stevens JL, Brown ML (2005). Insurance status and the use of guideline therapy in the treatment of selected cancers. *Journal of Clinical Oncology Official Journal of the*. Proc Am Soc Clin Oncol.

[13] Shi L (2001). Type of health insurance and the quality of primary care experience. Am J Public Health.

[14] Bisgaier J, Rhodes KV (2011). Auditing access to specialty care for children with public insurance. N Engl J Med.

[15] Kwok J, Langevin SM, Argiris A, Grandis JR, Gooding WE, Taioli E (2010). The impact of health insurance status on the survival of patients with head and neck cancer. Cancer.

[16] Groth SS, Al-Refaie WB, Zhong W, Vickers SM, Maddaus MA, D'Cunha J, Habermann EB (2013). Effect of insurance status on the surgical treatment of early-stage non-small cell lung cancer. Ann Thorac Surg.

[17] Bradley CJ, Dahman B, Bear HD (2012). Insurance and inpatient care: differences in length of stay and costs between surgically treated cancer patients. Cancer.

[18] Biswas T, Walker P, Podder T, Efird JT (2015). Effect of race and insurance on the outcome of stage I non-small cell lung Cancer. Anticancer Res.

[19] Potosky AL, Saxman S, Wallace RB, Lynch CF (2004). Population variations in the initial treatment of non-small-cell lung cancer. J Clin Oncol Off J Am Soc Clin Oncol.

[20] Bradley CJ, Given CW, Roberts C (2001). Disparities in cancer diagnosis and survival. Cancer.

[21] Sobin L, Gospodarowicz M, Wittekind C, Gospodarowitcz M, Sobin L, Gosporarowicz M (2009). International Union against Cancer (UICC): TNM classification of malignant tumours.

[22] Peterson ED, Delong ER, Masoudi FA, O'Brien SM, Peterson PN, Rumsfeld JS, Shahian DM, Shaw RE (2010). ACCF/AHA 2010 position statement on composite measures for healthcare performance assessment. J Am Coll Cardiol.

[23] Halpern MT, Ward EM, Pavluck AL, Schrag NM, Bian J, Chen AY (2008). Association of insurance status and ethnicity with cancer stage at diagnosis for 12 cancer sites: a retrospective analysis. Lancet Oncol.

[24] Greenberg ER, Chute CG, Stukel T, Baron JA, Freeman DH, Yates J, Korson R (1988). Social and economic factors in the choice of lung cancer treatment. A population-based study in two rural states. N Engl J Med.

[25] Kane S, Shaya F (2008). Medication non-adherence is associated with increased medical health care costs. Dig Dis Sci.

[26] Col N, Fanale JE, Kronholm P (1990). The role of medication noncompliance and adverse drug reactions in hospitalizations of the elderly. Arch Intern Med.

[27] Li Y, Wu Q, Xu L, Legge D, Hao Y, Gao L, Ning N, Wan G (2012). Factors affecting catastrophic health expenditure and impoverishment from medical expenses in China: policy implications of universal health insurance. Bull World Health Organ.

[28] Qiu P, Yang Y, Zhang J, Ma X (2011). Rural-to-urban migration and its implication for new cooperative medical scheme coverage and utilization in China. BMC Public Health.

[29] Lei X, Lin W: The new cooperative medical scheme in rural China: does more coverage mean more service and better health? Health Econ 2009, 18 Suppl 2(Supplement):S25. 10.1002/hec.1501 19551752

[30] Ruckdeschel JC, Finkelstein DM, Ettinger DS, Creech RH, Mason BA, Joss RA, Vogl S (1986). A randomized trial of the four most active regimens for metastatic non-small-cell lung cancer. J Clin Oncol.

[31] Lapar DJ, Bhamidipati CM, Mery CM, Stukenborg GJ, Jones DR, Schirmer BD, Kron IL, Ailawadi G (2010). Primary payer status affects mortality for major surgical operations. Ann Surg.

[32] Mcdavid K, Tucker TC, Sloggett A, Coleman MP (2003). Cancer survival in Kentucky and health insurance coverage. Arch Intern Med.

[33] Smith TJ, Penberthy L, Desch CE, Whittemore M, Newschaffer C, Hillner BE, Mcclish D, Retchin SM (1995). Differences in initial treatment patterns and outcomes of lung cancer in the elderly. Lung Cancer.

[34] Mokhles S, Nuyttens JJ, Maat AP, Birim Ö, Aerts JG, Bogers AJ, Takkenberg JJ (2015). Survival and treatment of non-small cell lung Cancer stage I-II treated surgically or with stereotactic body radiotherapy: patient and tumor-specific factors affect the prognosis. Ann Surg Oncol.

[35] Billmeier SE, Ayanian JZ, Zaslavsky AM, Nerenz DR, Jaklitsch MT, Rogers SO (2011). Predictors and outcomes of limited resection for early-stage non–small cell lung Cancer. J Natl Cancer Inst.

