# Supplementary material for: The effect of low insurance reimbursement on quality of care for non-small cell lung cancer in China: a comprehensive study covering diagnosis, treatment, and outcomes
Source: BMC Cancer. 2018 Jun 25;18:683. doi: 10.1186/s12885-018-4608-y (PMC6019825; doi:10.1186/s12885-018-4608-y)
Supplement: Supplementary file 1 — Table S1. Eligible definition of selected indicators. (DOCX 15 kb) [file 12885_2018_4608_MOESM1_ESM.docx]

**Additional file 1**

Additional file 1. Eligible definition of selected indicators.*

| Indicators | Eligible patients |
| --- | --- |
| Process-of-care indicators |  |
| 1.Skeletal scintigraphy (ECT) and brain MRI or CT | Clinical stage III patients with NSCLC, before initiation of combination therapy |
| 2.Pulmonary function test | NSCLC patients who underwent lung resection |
| 3.EGFR gene mutation test | Clinical stage IV patients diagnosed with NSCLC |
| 4.Adjuvant chemotherapy | Patients with stage IB and II NSCLC received lobectomy |
| 5.Recommended for ACT | Patients after curative resection for NSCLC with stages IA or IB disease |
| 6.Postoperative radiation therapy | Patients who had an incomplete surgical resection and a pathological stage I or II NSCLC |
| 7.Radiographic assessment of chemotherapy response | Patients with a stage IV or IIIB with malignant pleural effusion NSCLC who received chemotherapy |
| 8.First-line chemotherapy | Patients with a stage IV or IIIB with malignant pleural effusion NSCLC with normal organ function and good performance status |
| 9.Lobectomy | patients with a clinical stage IA NSCLC at presentation and no medical contraindications |
| 10.Surgical resection | patients with a clinical stage I or II NSCLC at presentation and no medical contraindications |
| 11.Combination therapy | Patient with locally advanced NSCLC and good performance status |
| Outcome-of-care indicators |  |
| 1. Postoperative complications | NSCLC patients who underwent lung resection |
| 2. Metastases | NSCLC patients who underwent lung resection |
| 3. in-hospital mortality | NSCLC patients who underwent inpatient treatment |
| 4. 2-year mortality | NSCLC patients who underwent inpatient treatment |
| 5. length of hospital stay | NSCLC patients who underwent inpatient treatment |

*Abbreviations: ECT and brain MRI or CT: skeletal scintigraphy and brain Magnatic Resonance Imaging or Computed Tomography, EGFR: epidermal growth factor receptor, ACT: Adjuvant chemotherapy, NSCLC: non-small cell lung cancer.
