# Supplementary material for: The effect of low insurance reimbursement on quality of care for non-small cell lung cancer in China: a comprehensive study covering diagnosis, treatment, and outcomes
Source: BMC Cancer. 2018 Jun 25;18:683. doi: 10.1186/s12885-018-4608-y (PMC6019825; doi:10.1186/s12885-018-4608-y)
Supplement: Supplementary file 2 — Table S2. Medical record questionnaire for non-small cell lung cancer patients. (DOCX 22 kb) [file 12885_2018_4608_MOESM2_ESM.docx]

**Additional file 2**

**Medical record questionnaire for non-small cell lung cancer patients***

**Demographic information and Tumor characteristics**

1.Patient ID: ;

2.Gender: ①male; ②female;

3.Age: ;

4.Annual household income: RMB;

5.Residence: ①urban; ②rural;

6. Primary payer status : ①Urban Resident Basic Medical Insurance (URBMI); ②Urban Employed Basic Medical Insurance (UEBMI); ③New Rural Cooperative Medical Scheme (NCMS); ④Others:____________;

7. Admission date: ; length of hospital stay: days;

8. Comorbidities: ①none; ②have:

| ①hypertension | ②diabetes mellitus | ③tuberculosis | ④bronchitis |
| --- | --- | --- | --- |
| ⑤pneumonia | ⑥tuberculous pleurisy | ⑦coronary heart disease | ⑧heart failure |
| ⑨myocardial infarction | ⑩angina | ⑪stroke | ⑫liver disease |
| ⑬historical cancer | ⑭others： | | |

9.Smoke: ①no; ②yes;

10.Family history of NSCLC: ①none; ②have;

11. Postoperative pathological report: ①none; ②have;

12.Primary lesion site: ①left; ②right; ③other;

13.Tumour size: __________cm;

14.Histological grade: ①high differential; ②moderately differential; ③low differential;

15.Histological classification: ①squamous carcinoma; ②adenocarcinoma; ③other;

17.Bronchial stump: ①negative; ①positive;

18. Lymphadenectomy: / (number of positive lymph nodes / number of removed lymph nodes);

Detailed lymphadenectomy information: ;

19.Distant metastases： ①no; ②yes;

20.Tumor stage:

①clinical stage: T______; N_____; M_____;

②pathological stage: T______; N_____; M_____;

21.EGFR gene mutation test: ①negative; ②positive; ③did not test.

**Inspection item**

| Item name | inspection |
| --- | --- |
| 1. brain Magnatic Resonance Imaging | ①no; ②yes; date： ； |
| 2. brain Computed Tomography | ①no; ②yes; date： ； |
| 3. skeletal scintigraphy | ①no; ②yes; date： ； |
| 4. bronchoscope | ①no; ②yes; date： ； |
| 5. pulmonary function test | ①no; ②yes; date： ； |
| 6. PET-CT | ①no; ②yes; date： ；； |

**Therapy**

1.Combination therapy: ①no; ②yes;

2.Surgery: ①no; ②yes;

Date of surgery: (yyyy/mm/dd);

Surgeon: ;

3.Preoperative communication: ①no; ②yes; ③not recorded

4.Surgical procedures: ①lobectomy; ②wedge resection: ③pneumonectomy; ④ exploratory thoracotomy

5. Recommended for postoperative Adjuvant chemotherapy: ①no; ②yes;

6. Postoperative adjuvant chemotherapy: ①no; ②yes;

Chemotherapist: ;

Department of chemotherapy: ①internal medicine department; ②radiology department; ③surgery department; ④other ;

Periodic number: ;

Scheme: ;

Date:

7.Neoadjuvant chemotherapy: ①no; ②yes;

periodic number:

8.First-line chemotherapy: ①no; ②yes;

Periodic number: ;

Scheme: ;

Date: ;

9.Postoperative radiation therapy: ①no; ②yes;

**Outcome**

Postoperative complication: ①no; ②yes:

| ①pneumonia | ②pulmonary atelectasis | ③respiratory failure or even ARDS |
| --- | --- | --- |
| ④bronchospasm | ⑤respiratory insufficiency | ⑥pulmonary artery embolism |
| ⑦arrhythmia | ⑧postoperative blood chest/ empyema. | ⑨postoperative hypotension |
| ⑩cerebral infarction | ⑪others： | |

The patients were informed of the follow-up plan at discharge: ①no; ②yes:

| 1.in-hospital mortality | ①no; ②yes; date： ； |
| --- | --- |
| 2.postoperative recurrence | ①no; ②yes; date： ； |
| 3.postoperative metastasis | ①no; ②yes; date： ； |
| 4.death after discharge | ①no; ②yes; date： ； |

*To guarantee the validity and reliability of the questionnaire, we conducted a pilot test. During the data collection process, regular correspondence was maintained with those compiling the data to identify any ambiguities or deficiencies in the information collection to facilitate timely modification and accelerate the process of data extraction. Following the data collection, 5% of the records were randomly selected for a secondary data collection using methods identical to the first data collection, and the test-retest reliability was high (up to 95%).
